# Severe acute hepatitis of unknown etiology in a large cohort of children

**DOI:** 10.1097/HC9.0000000000000272

**Published:** 2023-09-27

**Authors:** Sagar Mehta, Tomisin John, Jordan J. Feld, Hemant Shah, Nisa Mullaithilaga, Aaron Campigotto, Karen Leung, Binita M. Kamath, Simon C. Ling, Michelle Science, Vicky L. Ng

**Affiliations:** 1 Department of Paediatrics, Division of Gastroenterology, Hepatology and Nutrition, Hospital for Sick Children, University of Toronto, Toronto, Ontario, Canada; 2 Department of Paediatrics, Transplant and Regenerative Medicine Centre, Hospital for Sick Children, University of Toronto, Ontario, Canada; 3 Toronto Centre for Liver Disease, University Health Network, University of Toronto, Toronto, Ontario, Canada; 4 Department of Paediatrics, Division of Infectious Diseases, The Hospital for Sick Children, University of Toronto, Toronto, Ontario, Canada; 5 Department of Laboratory Medicine, Division of Microbiology, University of Toronto, Toronto, Ontario, Canada

## Abstract

**Background:**

We evaluated the proportion, clinical features, and outcomes of previously healthy children presenting to a large Canadian quaternary pediatric center with severe acute hepatitis of unknown etiology.

**Methods:**

All patients with serum alanine aminotransferase (ALT) > 500 U/L or aspartate aminotransferase (AST) > 500 U/L between June 1, 2018, and May 31, 2022, at The Hospital for Sick Children, were identified. Subjects with only AST > 500 U/L were excluded. Clinical characteristics, investigations, and outcomes for patients without clear etiology for ALT > 500 U/L (severe acute hepatitis of unknown etiology) for our study period and from October 1 to May 31 of each year 2018–2021 were reviewed.

**Results:**

Of 977 patients with ALT/AST> 500 U/L, 720 had only ALT > 500 U/L. We excluded age below 6 months (n = 99) or above 16 years (n = 66), known pre-existing liver conditions (n = 66), and ALT > 500 U/L in already admitted patients (n = 151). Among the remaining 338 children with ALT > 500 U/L at presentation, an etiology was identified in 303 subjects. 33 (9.8%) children [median age 6.1 y (range 0.5–15.5); 61% male] were confirmed as severe acute hepatitis of unknown etiology. Twenty patients (60.6%) were tested for blood adenovirus by PCR, and 1 (5%) was positive (serotype B7). Liver tissue specimens from 18 patients revealed no evidence of viral inclusions or adenovirus. Twelve (36.3%) presented with pediatric acute liver failure, with 8 (24.2%) requiring liver transplantation. There were no deaths. Hepatitis-associated aplastic anemia occurred in 5 (15%) patients.

**Conclusions:**

Of children presenting with severe acute hepatitis to a quaternary children’s hospital over a 48-month period, 9.8% had unknown etiology with no change over time. Liver transplantation remains an important treatment strategy for those presenting with pediatric acute liver failure phenotype. The frequency of cases associated with human adenovirus infection was noncontributory.

## INTRODUCTION

Following concern raised in April 2022 by public health agencies worldwide, over 1000 cases of severe acute hepatitis of unknown etiology (SAH-UE) in children have been reported to the World Health Organization (WHO) and the Centers for Disease Control and Prevention (CDC).^1,2^ It is not uncommon to see children presenting with elevated liver transaminase levels, including the most severe phenotype of pediatric acute liver failure due to indeterminate etiology (PALF-ID).^3^ The recent use of genomic and infectious investigations has suggested a helper virus theory to accompany the initial focus on adenovirus.^4^ However, the lack of historical monitoring, initial use of varying case definitions, and incomplete evaluation have added to concern about the actual disease burden amid anxieties from a world still recovering from the COVID-19 pandemic.^5–7^ To date, the proportion of SAH-UE presenting to quaternary pediatric liver care centers remains unknown. Our aim was to address this knowledge gap using a modified WHO definition.

This study first identified all children presenting with alanine aminotransferase (ALT) or aspartate aminotransferase (AST) > 500 U/L at a large quaternary hospital in Canada, before targeting just those with ALT>500 U/L. Our primary objective was to determine the proportion of children presenting to a large pediatric institution with severe acute hepatitis, who remained without a specific etiology after extensive workup (SAH-UE). The secondary objectives were to evaluate the outcomes of those children with SAH-UE and explore the potential etiological role of adenovirus and severe acute respiratory syndrome coronavirus-2 (SARS-CoV-2) infection.

## METHODS

This was a retrospective single-center cohort study at the Hospital for Sick Children (SickKids), Toronto, Canada. The study period of June 1, 2018, to May 31, 2022, was chosen to ensure adequate time periods for prepandemic and postpandemic and to account for possible seasonality.

### Study participants

All children (under 18 y of age) with an ALT or AST > 500 U/L at the time of first presentation to our hospital were identified by our institutional electronic medical record team (Epic Systems, Verona) using the WHO definition of probable case of severe acute hepatitis (Table 1). Patients with only ALT > 500 U/L between ages 6 months and 15.99 years were reviewed. We excluded hospitalized patients who developed ALT rise of >500 U/L after admission and those who had a known pre-existing liver condition for which SickKids Liver clinic follow-up was already in place (eg, Alpha-1 antitrypsin deficiency, chronic hepatitis B/C, autoimmune hepatitis, Wilson disease, fatty liver, or DILI) (Supplemental Table S1A, http://links.lww.com/HC9/A538). Further excluded were patients i) without Hepatitis A, B and C serology testing, ii) with newly diagnosed childhood liver diseases and iii) those in whom a specific etiology (e.g., malignancy, ischemic or vascular events, metabolic or genetic disorders, drug related hepatitis or systemic diseases, including first elevated ALT level was noted immediately post a surgical (cardiac, major trauma) procedure) was attributable to the acute elevation in ALT (Supplemental Table 1b, http://links.lww.com/HC9/A538).

**TABLE 1:** WHO working case definition

Confirmed case	Probable case	Epidemiologically linked case
Not defined	Age ≤16 and Since October 1, 2021, and Acute hepatitis (non-hep A–E) and AST or ALT > 500 U/L	Any age who is a close contact of a probable case Since October 1, 2021 Acute hepatitis non-hep A–E

Abbreviations: ALT, alanine aminotransferase; AST, aspartate aminotransferase.

Patients presenting to the Emergency Room or referrals from other hospitals were triaged based on clinical and laboratory parameters. Pediatric acute liver failure (PALF) was defined as any child presenting with evidence of acute and severe hepatic dysfunction without any underlying chronic liver disease AND an uncorrectable international normalized ratio (INR) ≥2.0 without encephalopathy or ≥1.5 with encephalopathy, as per the definitions provided by the National Institute of Health (NIH)–funded Pediatric Acute liver Failure Study Group (PALFSG).^8–10^ Workups for severe acute hepatitis and PALF were based on age-appropriate clinical scenarios (Supplemental Table S2, http://links.lww.com/HC9/A538). The start of the COVID-19 period was January 24, 2020, following the first positive case recorded in Canada.^11^

Hepatitis E is not common in Canada and was not routinely tested in all patients. Quantitative adenovirus PCR was performed with the RealStar PCR kit 1.0 (Altona, Hamburg, Germany) in plasma samples. Stool and nasopharyngeal (NP) specimens were tested as a panel for multiple viruses including adenovirus 40/41 using Allplex GI-Virus assay (Seegene, South Korea) and Allplex Respiratory Panel 2 (Seegene), respectively. Adenovirus genotyping was performed through sequencing of the hypervariable region of the hexon gene with subsequent BLAST analysis using the GenBank database.^12^ Histopathology was reviewed from liver biopsy samples (if available) or an explant after liver transplant. Patients with PALF requiring liver transplantation (LT) were listed as 4F or 3F as per the Provincial listing algorithm by Ontario’s Organ and Tissue Donation Agency.^13^

### Data sources

Patient demographic, biochemical, radiographical, and histopathological (as available) data were reviewed by extensive chart review and stratified as “specific etiology identified” versus “specific etiology not identified (SAH-UE)” by coauthors (Aaron Campigotto, Tomisin John, Simon C. Ling, Nisa Mullaithilaga, Sagar Mehta, Vicky L. Ng, and Michelle Science). Clinical presentation, diagnostic workup, and outcomes were retrospectively collected for the patients with SAH-UE using a study data collection form.

### Analysis of trend of cases

Trends in the number of patients with hepatitis (ALT > 500 U/L), PALF-ID, and LT for PALF-ID between October 1, 2021, and May 31, 2022, were compared to October 1-May 31 for each of the years 2018-2019, 2019-2020, and 2020-2021, respectively. PALF-ID was considered as the severe phenotype of severe acute hepatitis in children.

### Statistical analysis

Descriptive analysis (continuous data were expressed as the median and range, and dichotomous data were expressed as frequency and percentage) was used to present the demographics and clinical characteristics of the cohort.

### Research ethics board

The study was conducted in accordance with both the Declarations of Helsinki and Istanbul and approved by the Research Ethics Board (REB # 1000079986) of the Hospital for Sick Children, with an approved waiver of informed consent.

## RESULTS

### Study population

**FIGURE 1:**
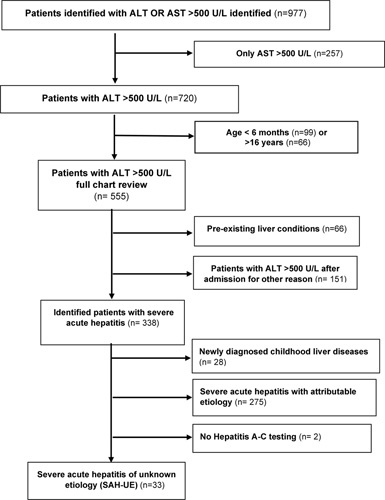
Consort diagram detailing the process of identification of cases with severe acute hepatitis of unknown etiology. Abbreviations: ALT, alanine aminotransferase; AST, aspartate aminotransferase.

Our initial query identified a total of 977 patients under the age of 18 years with ALT or AST > 500 U/L within the study period. There were 257 patients with only AST > 500 U/L, who were excluded from further review. Figure 1 provides a flowchart of the systematic exclusion of patients from further analyses. Of 338 patients presenting with ALT > 500 U/L for the first time, an identifiable cause included a newly diagnosed chronic liver condition (n = 28), acute hepatic (n = 77), and nonhepatic (n = 198) etiologies (Supplemental Table S1A, B, http://links.lww.com/HC9/A538). Two patients found without hepatitis A, B, and C testing were excluded. The final study cohort is, thus, comprised of 33 (9.8%) patients in whom an etiology for ALT > 500 U/L at presentation remained unexplained despite extensive investigations (SAH-UE).

### Identification of SAH-UE cases

**FIGURE 2:**
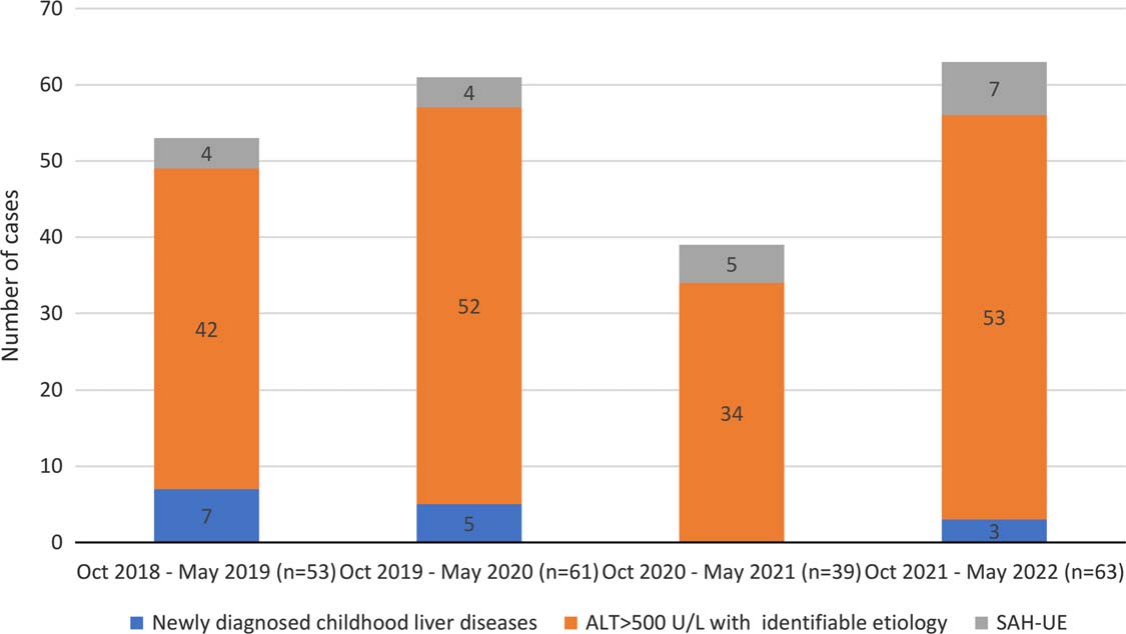
Number of cases of newly diagnosed childhood liver conditions, severe hepatitis of identifiable etiologies, and SAH-UE. Abbreviations: ALT, alanine aminotransferase; SAH-UE, severe acute hepatitis of unknown etiology.

Figure 2 shows the proportion of SAH-UE cases, ALT >500 U/L with attributable etiologies and newly diagnosed liver conditions between October 1 and May 31 for the years 2018-2019, 2019-2020, 2020-2021, and 2021-2022. Between October 2021 and May 2022, 7/63 (11.1%) cases of SAH-UE were identified. This is comparable to 4/53 (7.5%) cases in 2018-2019, 4/61 (6.5%) in 2019-2020, and 5/39 (12.8%) in 2020-2021, with stable numbers of PALF-ID patients, at 0–3 cases per period. The number of LTs performed for PALF-ID patients was 0-3 per observation period. These numbers are consistent with trends in our program over 3 decades (V.L. Ng, written communication, May 2023).

### Demographics and clinical characteristics of study cohort

Table 2 provides the baseline characteristics of the 33 participants in the study cohort of SAH-UE patients. The median age at first presentation was 6.1 years (range 0.5–15.5), with 42% presenting between the ages of 6 months and 5 years; 61% were male and 40% were of Asian background. Over two-thirds (67%) of SAH-UE cases were referred to our center with the first documented ALT > 500 U/L before transfer to SickKids. Four (12%) patients were investigated on an outpatient basis.

**TABLE 2:** Baseline demographic, clinical, and laboratory features of children with severe acute hepatitis of unknown etiology SAH-UE (n = 33)

Variables		SAH-UE cohort
Median age, y (range)	n=33	6.09 (0.53–15.55)
0.5–5, n (%)	—	14 (42.5)
5–12, n (%)	—	11 (33.5)
12–<16, n (%)	—	8 (24)
Sex: male, n (%)	—	20 (61)
Ethnicity, n (%)	n=33	—
Asian	—	13 (40)
White	—	3 (9)
Other [Indigenous-4, Black-2, Hispanic-2, Arab-1]	—	9 (27)
Not reported	—	8 (24)
Symptoms/signs, n (%)	n=33	—
Jaundice	—	20 (61)
Abdominal pain	—	16 (48)
Fever	—	20 (61)
Vomiting	—	17 (52)
Loss of appetite	—	16 (48)
Diarrhea	—	7 (21)
Upper respiratory symptoms	—	11 (33)
Rash	—	6 (18)
First ALT>500 U/L at outside hospital, n (%)	—	22 (67)
Pediatric acute liver failure (PALF) phenotype, n (%)	—	12 (36.3)
Investigation in outpatient clinic (n)	—	4
Liver transplantation, n (%)	—	8 (24.2)
Death	—	0
Hepatitis-associated aplastic anemia (HAAA), n (%)	—	5 (15)
SARS-CoV-2 vaccination	n=24	6 (25)
Laboratory parameters	No. tested	Median (range)
ALT, U/L Presentation	33	1490 (60–8892)
Peak	—	1597 (526–10,000)
Interval from presentation to peak, d^a^	—	0 (0–6)
AST, U/L presentation	33	1470 (140–10,000)
Peak	—	1807 (185–10,000)
Conjugated bilirubin, µmol/L	33	—
Presentation	—	66 (0–285)
Peak	—	108 (0–338)
Interval from presentation to peak, d	—	2 (0–16)
INR		
Presentation	33	1.4 (1–4.2)
Peak	—	1.5 (1–9)
Interval from presentation to peak, d	—	1 (0–18)
Albumin, g/L	32	37 (23–50)
White blood cells (WBC) 10^9^ /L	33	5.75 (0.85–15.5)
Infectious etiology tests	No. tested	No. positive test
Blood PCR		
Adenovirus	20	1 positive
Cytomegalovirus	28	1 positive
Epstein-Barr virus	29	1 positive
Human herpes virus 6	28	1 positive
Herpes simplex virus-1^b^	18	1 positive
Enterovirus	11	None
Varicella zoster virus	17	None
Stool PCR	9	None
Nasopharyngeal PCR		
Adenovirus	12	None
Enterovirus	12	2 positives
Human Coronavirus OC43	12	1 positive
SARS-CoV-2 PCR	19	None
Urine adenoviral PCR	1	Negative

a Interval to peak ALT (n = 22) calculated in non-liver transplant patients.

b Co-existing influenza A in NP specimen.

Abbreviations: ALT, alanine transaminase; AST, aspartate transaminase; INR, international normalized ratio; SAH-UE, severe acute hepatitis of unknown etiology; SARS-CoV-2, severe acute respiratory syndrome coronavirus-2.

Jaundice was the most common presenting symptom seen in 61% of our study cohort. Other symptoms included fever (61%), vomiting (55%), abdominal pain (45%), diarrhea (21%), and upper respiratory symptoms (38%). Clinical examination findings at presentation included jaundice (62%), hepatomegaly (48%), splenomegaly (2%), ascites (3%), and hepatic encephalopathy (HE) (7%).

### Laboratory parameters

Median ALT was 1490 U/L (range 60–8892 U/L), with peak ALT (median 1597 U/L, range 526–10,000) occurring at a median interval of 1 day (range 0–15) from the first presentation. Median values at presentation for AST, conjugated bilirubin, and INR were 1470 (range 145–10,000) U/L, 66 (range 0–285) µmol/L, and 1.4 (range 1–4.2), respectively (Table 2). Low white blood cells (WBC < 4 × 10^9^/L) were noted in 9 (27.2%) patients and thrombocytopenia (platelet count under 150 × 10^9^/L) in 4 (12%) children. Serum ferritin levels were available in 29/33 children and ranged between 22.6 and 26,473 µg/L (normal range 5.3–99.9 µg/L). In 2 instances, serum ferritin levels were as high as 6574 and 26,473 ug/L with a negative Hemophagocytic lymphohistiocytosis workup.

Hepatitis E IgM antibody testing in 21/33 children was all negative. Results of serum immunoglobulin levels were available in 31/33 children, with higher than normal values in only 1 patient. Results for serum autoantibodies were available in 30 (91%) SAH-UE children, with 50% positivity in antinuclear antibody (n = 15) and 27% antismooth muscle antibody (n = 8), but no patients were positive for anti–liver kidney microsomal antibody. Available liver histology in 8/19 patients with antinuclear antibody or antismooth muscle antibody positivity did not show evidence of autoimmune hepatitis including the patient with elevated IGG level. Among 24 patients with SAH-UE with age above 5 years, serum ceruloplasmin values available in 22 (91.7%) children were all in the normal range. Alpha1-antitrypsin levels and celiac serology testing were available in 27 and 21 children, respectively, which were in the normal range. Results from 16 (48.5%) blood acetaminophen levels and 18 (54.5%) available urine toxicology screens were all within the normal range.

### Workup for infectious etiologies for 33 SAH-UE cases

During the COVID-19 period, 19/24 patients were tested for SARS-CoV-2 PCR in a NP swab, and all were negative; serology (anti-Spike IgG) was reactive in 3/5 (60%) of tested patients. 6/24 (25%) children (median age 13 y, range 5.3–14.83 y) had received either 1 or 2 doses of the COVID-19 vaccine. Other pathogen testing was variable. Cytomegalovirus (CMV) PCR in blood was positive (5800 IU/mL) in 1 patient whose CMV IgM was negative and IgG was equivocal. In another patient, Epstein-Barr virus (EBV) was detected by PCR in blood, but EBV serology showed negative EBV VCA IgM antibody. Other positive PCR testing results included human herpes virus 6 (1/28) and herpes simplex virus (HSV-1) (1/18) in blood, while enterovirus (2/12) and human coronavirus OC43 (1/12) in NP specimens (Table 2).

### Adenoviral testing

Testing for adenovirus was performed in 21 (64%) cases of SAH-UE. This included PCR testing of blood (n = 20), NP samples (n = 12), stool samples (n = 9), and urine (n = 1). All 12 (100%) patients with PALF presentation had adenovirus PCR testing of blood. Respiratory symptoms were present in 41% who underwent NP tests, while diarrhea was present in 25% who had stool testing. Adenoviral DNA (<1000 copies/mL, serotype B7) was detected in the blood of a 1.65-year-old male who presented with PALF requiring LT 6 days after referral. Adenovirus DNA was not detected in this child’s stool or urine, liver biopsy, or in the explanted liver. Between October and May of 2021-2022, 4/7 patients had blood adenovirus PCR tested compared to two-fourth, three-fourth, and two-fifth children during the same period in 2018-2019, 2019-2020, and 2020-2021, respectively. This was not comparable statistically because of small numbers in our cohort.

### Histology from liver tissue

Liver tissue was examined in 18 patients (14 needle biopsies and 8 explants). Nine patients had moderate to severe hepatitis, and 12 had severe hepatocyte loss. None had significant fibrosis or the presence of viral inclusion bodies. Immunohistochemistry was performed for adenovirus (7/18), CMV (9/18), HSV-1, and HSV-2 (5/18), while EBV in situ hybridization was done on 6/18; all were negative. Adenoviral PCR was performed in 3 tissue specimens; all were negative. One child had concurrent biopsy tissue PCR positive for EBV and human herpes virus 6, while one had only positive for human herpes virus 6 by PCR.

### Outcome

Twelve of the 33 (36.3%) of SAH-UE patients presented in PALF. Two patients presented with HE and serum ammonium levels of 15 and 91 (µmol/L), respectively, which peaked at 32 and 120 (µmol/L), respectively. Of the remaining 6 patients, 5 eventually developed HE after admission. The median interval from admission to LT for 8 of the 12 PALF patients was 6 days (range 3–9 d). One patient had positive blood HSV-1 PCR and co-infection with influenza A without evidence of viral inclusion on explant tissue examination. Spontaneous recovery of the native liver occurred in 4 of 12 PALF patients. While no deaths were recorded, progression to the need for life-saving LT occurred in 8 (24.2%) SAH-UE patients, all with PALF phenotype. Spontaneous recovery of the native liver occurred in 25 (75.8%), including 4/12 (33%) children presenting with PALF. Patients requiring LT were younger (4.12 y, range 1.3–14.56 y) and more commonly males (87.5%).

The median ALT was 45 (range 12–1239) at 61 days (range 2–611 d) from the first presentation. At the last follow-up of a median of 123 days (range 16–611 d), 40% had ALT <40 U/L.

Five (5/33) children (male-3, age range 1.3–13.89 y) were diagnosed with hepatitis-associated aplastic anemia, at a median time of 60 days (range 2–156 d) from the first SAH-UE presentation. Three (60%) eventually required bone marrow transplantation and are alive at the time of writing this report (Table 3).

**TABLE 3:** Characteristics of patients with hepatitis-associated aplastic anemia (HAAA) in the SAH-UE cohort

Pts	Age, y	Sex	PALF	Potential pathogen	Nadir platelet count (×10^9^/L)	Nadir WBC (×10^9^/L)	Time (d) to aplastic anemia Dx	Liver transplant	Bone marrow transplant	Status
1	1.3	Male	Yes	No	54	0.56	53	Yes	No	Alive
2	8.16	Female	No	No	4	0.02	156	No	Yes	Alive
3	13.89	Female	No	No	1	0.20	60	No	Yes	Alive
4	12.27	Male	Yes	No	51	2	117	No	Yes	Alive
5	4.41	Male	No	Yes (Rhinovirus on NP swab)	2	0.10	2	No	No	Alive

Abbreviations: NP, nasopharyngeal; PALF, pediatric acute liver failure; WBC, white blood cell.

## DISCUSSION

The number of children presenting with acute hepatitis without clear explanation has not been monitored historically. It remains unclear whether worldwide case counts of >1000 over a 3-month period in 2022 represented a true increase or is reflective of heightened awareness and increased reporting.^1,5^ This study addresses this gap in knowledge by seeking to identify all children presenting with ALT > 500 U/L with eventual known and unknown etiologies, including PALF. Among children presenting with acute hepatitis over a 4-year period to our large pediatric quaternary institution in Canada, despite extensive investigations, 33 (9.8%) had no clear etiology. Among these 33 SAH-UE cases, 12 (36.3%) children presented in or progressed to PALF. All patients remain alive with no deaths. LT was required in about one-quarter of the subjects. Aplastic anemia was diagnosed in 5 children, with 1 occurrence diagnosed post-transplant.

To date, the published literature describes variation in the reported incidence of cases since October 2021, attributed in part to broad case definitions with variable interpretations, with different testing and case-finding practices.^5^ The number of SAH-UE cases in our study may be lower compared to previous reports for several reasons. First, we used ALT to identify patients with hepatitis as it is more liver-specific, thus excluding patients presenting only with AST > 500 U/L.^14^ Second, we excluded children aged <6 months from our analysis who present with unique etiologies of severe hepatitis, such as birth asphyxia/hypoxic-ischemic encephalopathy, congenital infections, congenital malformations, multisystemic disease, sepsis, and postsurgical causes. We also systematically excluded patients with newly identified childhood liver diseases, such as autoimmune hepatitis, and those with a nonhepatic explanation of ALT > 500 U/L from the study cohort, which may inflate the numerator of cases with SAH-UE.

Using the same definition, the proportion of SAH-UE cases presenting between the months of October and May within 2020-2021 and 2021-2022 was 12.8% and 11.1%, respectively. While these numbers are slightly higher than in previous years, the small number of cases in our study cohort precluded meaningful statistical analysis. We did not see an increase in isolated LT numbers for PALF-ID diagnoses. The recent increase in SAH-UE cases could be secondary to isolation and containment measures, and lower rates of vaccination during the COVID-19 pandemic, resulting in an altered immune response to infections as hypothesized by other authors.^15–18^

Guidance on the evaluation of severe acute hepatitis is available in the published literature.^6,19^ Practically, the finding that a diagnostic workup is incomplete can be the result of multiple influences. These include the severity of hepatitis on presentation, the presence of comorbidities, and consultation with the subspecialist. In our cohort, there were no differences in investigations performed in patients admitted under the general pediatric teams or subspecialty wards, possibly due to the creation of clinical pathways for “elevated liver transaminase levels” within the electronic medical record. However, 2 patients with less thorough workups did not have a pediatric gastroenterologist/hepatologist involved in their care, underscoring the value of early consultation. In a review, 49% of indeterminate adult acute liver failure patients who were reevaluated had a defined etiology after further testing.^20^

Nearly one-third of our SAH-UE cohort presented with acute liver failure. These children presenting with evidence of an acute liver injury and worsening of initial INR >2.0 after i.v. vitamin K therapy are the most concerning SAH-UE phenotype. One hundred percent of those requiring LT were children presenting with PALF underscores the importance of timely referral to a liver transplant center. Although most children with SAH-UE recovered with their native livers, LT was required in 24.2% of patients, in contrast to the 5% reported in the earlier cohorts.^6^ This may in part be attributed to a referral bias, given that we are 1 of just 3 pediatric LT programs in Canada. In those who did not have LT, the last known ALT level, when available, was <40 U/L in about 45% of children.

Hepatitis-associated aplastic anemia was diagnosed in over 15% of our cohort. This is higher than the reported 6.6% prevalence of ALF in the published literature and may not be that different given the small absolute numbers.^21,22^ Hematopoietic stem cell transplantation was required in 60% of our patients, which is higher than the prevalence of 25% previously reported by colleagues in the United Kingdom,^21^ highlighting the severity of the disease in our cohort. Our data support close monitoring of this complication and for early involvement of hematology experts for prompt management.

Reports of 100% of cases in Alabama and >50% of cases reported in the United Kingdom and by the European Centre for Disease Prevention and Control (ECDC) had blood adenovirus identified by real-time PCR; however, adenovirus was not confirmed to be directly present in liver biopsies.^6,16,23–25^ The presence of adeno-associated virus detected using metagenomics in blood and liver specimens of children with severe acute hepatitis suggests a possible role in causing acute hepatitis in genetically susceptible children.^26–29^ These findings contrast the historical notion that adenovirus hepatitis is rare in immunocompetent children.^30^ Adenovirus was tested in less than two-thirds of our cohort. However, as per our hospital protocol, blood adenovirus PCR testing was done in 100% of PALF patients, of which only one was positive. Jagadisan et al^31^ showed adenoviremia in 100% of PALF-ID cases and a significant number of hepatitis cases without evidence of adenovirus in liver tissues in the recent outbreak, which is in contrast to 2020-2021, owing to increased background adenoviral infection rates. In our study, the histopathology review of all diagnostic and explant liver biopsies was negative for viral inclusion bodies and adenovirus, supporting the theory of an unidentified trigger causing severe inflammation of the hepatocytes.

During the pandemic, none of the tested patients (79%) were positive for SARS-CoV-2 PCR. A quarter of the children were vaccinated, and some showed antibodies against SARS-CoV-2. There is no evidence of a link between the SARS-CoV-2 vaccine with severe acute hepatitis in children, and most cases to date have been among children less than 5 years of age and who were not yet vaccine-eligible or who had not received a SARS-CoV-2 vaccine.^6,23^

In our identified cases, several other pathogens were identified in bodily fluids; however, their significance in causing severe hepatitis or acute liver failure is argued. A recent report from the Cincinnati group showed increased numbers of non-A-C hepatitis comprising new viruses (adenovirus > SARS-CoV-2 > EBV/CMV or mixed infections) in 2021-2022 compared to historical cohorts suggesting increased circulating pathogens. However, there was no evidence in the liver tissue, limiting conclusions about causality.^32^

We acknowledge some limitations to our study. Because of the retrospective design, missing clinical, laboratory, and follow-up data exist in our study. For the same reasons, incomplete evaluation of cases may overestimate the numerator. In addition, workup may not be uniform for all patients over the study period. The changes in our protocol after the recent spotlight on SAH-UE may have led to increased vigilance and testing. Another limitation is that our study period accounts for only the first 5 months of 2022, contributing to a lower number of adenoviral tests compared to other reports.

In conclusion, of children presenting with severe acute hepatitis to a large quaternary care pediatric institution in Canada, 9.8% had no identifiable etiology, which was relatively stable over time with no clear increase. We did not observe an increase in the number of indeterminate PALF, which is the most severe phenotype of acute hepatitis in children. Although testing was done in only 64% of our SAH-UE cohort, human adenovirus 41 was not observed to be of relevance. Multicenter prospective studies are needed to ascertain the role of adenovirus or other infectious etiology. Most children recovered with supportive care; however, reports of LT and death in other centers underscore the importance earliest referral of those with PALF phenotype.

## Supplementary Material
