# Supplementary material for: Severe acute hepatitis of unknown etiology in a large cohort of children
Source: Hepatol Commun. 2023 Sep 27;7(10):e0272. doi: 10.1097/HC9.0000000000000272 (PMC10531196; doi:10.1097/HC9.0000000000000272)
Supplement: Supplementary file 1 [file hc9-7-e0272-s001.docx]

**Supplementary Table 1a**: Excluded patients with pre-existing and new liver diseases

| **Pre-existing liver disease** | **No.** | **Newly diagnosed childhood liver conditions** | **No.** |
| --- | --- | --- | --- |
| Biliary atresia, post Kasai surgery | 22 | Autoimmune hepatitis | 10 |
| Metabolic liver disease | 15 | Cholelithiasis and/or choledocholithiasis | 7 |
| Tumor | 9 | Acute cholecystitis | 2 |
| Alagille syndrome | 5 | Glycogen storage disorders | 2 |
| Post-liver/multivisceral transplant | 4 | Suspected primary sclerosing cholangitis | 2 |
| Cholangitis | 3 | Choledochal cyst | 1 |
| Acute cholecystitis/gall stone | 2 | Non alcoholic fatty liver/NASH with suspected medication adverse event | 1 |
| Glycogen storage disorders | 2 | NASH with limb girdle myopathy | 1 |
| Autoimmune hepatitis | 1 | Tumor- Hepatoblastoma | 1 |
| Congenital hepatic fibrosis | 1 | Wilson disease | 1 |
| Non alcoholic fatty liver/NASH | 1 | **Total** | **28** |
| PFIC | 1 |  |  |
| **Total** | **66** |  |  |
| NASH- Non alcoholic steatohepatitis, PFIC- Progressive familial intrahepatic cholestasis | | | |

**Supplementary Table 1b**: Severe hepatitis with an identifiable etiology

| **Etiologies** | | **No** |
| --- | --- | --- |
| **A.** | **Hepatic based etiology** | **n=77** |
|  | Infection |  |
|  | Hepatotropic viruses |  |
|  | Hepatitis A | 14 |
|  | Hepatitis B | 1 |
|  | Hepatitis E | 1 |
|  | Other infective etiologies |  |
|  | Adenovirus | 1 |
|  | CMV | 1 |
|  | EBV | 13 |
|  | Enterovirus | 2 |
|  | HSV-1 | 1 |
|  | Influenza | 4 |
|  | Rotavirus | 1 |
|  | RSV | 1 |
|  | SARS-CoV-2 | 1 |
|  | Bacterial sepsis | 4 |
|  | Malaria | 2 |
|  | Immunological | 1 |
|  | Hemophagocytic lymphohistiocytosis | 5 |
|  | Toxic/Drugs | 24 |
| **B.** | **Non-hepatic based etiology** | **n=198** |
|  | Cardiac condition | 6 |
|  | Endocrinological | 2 |
|  | Gastroenterological | 10 |
|  | Genetic | 3 |
|  | Hematological | 15 |
|  | Malignancy | 80 |
|  | Miscellaneous | 6 |
|  | Nephrological | 3 |
|  | Neuromuscular disease | 10 |
|  | Post-surgical | 3 |
|  | Rheumatological | 15 |
|  | Systemic | 26 |
|  | Trauma | 19 |
|  | **Total** | **275** |

| **Supplementary Table 2**: Investigations for severe acute hepatitis and pediatric acute liver failure | |
| --- | --- |
| Infectious etiology | Blood serology- Hepatitis A-E, non-hepatotropic viruses (CMV, EBV, Varicella, Parvovirus, HSV), SARS-CoV-2 antibodies  Blood PCR: CMV, EBV, HHV-6, Varicella, HSV, Adenovirus, Enterovirus  Nasal/throat- respiratory virus testing, SARS-CoV-2 PCR  Stool tests- stool viral PCR |
| Autoimmune hepatitis | Serum immunoglobulin G, antinuclear antibody, anti-smooth muscle antibody, anti-liver kidney microsomal antibody |
| Metabolic disease | Serum ceruloplasmin, 24-hour urine copper, ammonia, lactate, plasma amino acid level, urine organic acid profile, alpha-1-antitrypsin, |
| Toxins | blood and urine toxicology screen |
| Other: | Celiac screening, thyroid stimulating hormone, Ferritin, creatine kinase |
